# A reproducible U-net workflow for SEM/BSE bright-particle quantification

**DOI:** 10.1016/j.mex.2026.104134

**Published:** 2026-09-02

**Authors:** Ibrahim M. Ibrahim, Geoffrey Will, Stuart Bell

**Affiliations:** a School of Mechanical, Medical, Process Engineering, Faculty of Engineering, Queensland University of Technology (QUT), Qld, Australia; b Commonwealth Scientific and Industrial Research Organization (CSIRO), Melbourne, Australia; c School of Science, Technology and Engineering, University of the Sunshine Coast, Qld, Australia

**Keywords:** SEM/BSE image analysis, U-net segmentation, Duplex stainless steel, Bright-particle quantification, Reference mask generation, Connected-component analysis, Microstructure segmentation, Deep learning, Semantic segmentation, CNN

## Abstract

Thermal exposure of metallic alloys can promote the precipitation of deleterious secondary particles that degrade mechanical and corrosion performance, making their quantitative characterisation from SEM/BSE micrographs an important step in microstructural assessment. Quantifying precipitate regions in SEM/BSE micrographs of thermally aged steels can be difficult when image contrast, particle size, and particle connectivity change across conditions. This method describes a reproducible workflow for generating reference bright-particle masks, training a lightweight U-Net segmentation model, and converting predicted masks into conventional microstructural descriptors. The workflow combines contrast-enhanced preprocessing, condition-aware reference mask generation, image-level data splitting, overlapping patch extraction, supervised U-Net training, fixed-threshold inference, and connected-component analysis. It is intended for SEM/BSE datasets where bright regions are used as image-based indicators of Cr/Mo-rich deleterious particles, while recognising that crystallographic phase identification requires complementary evidence. In the present application, the reported performance metrics quantify agreement with threshold-derived reference masks on held-out micrographs; they should not be interpreted as validation against independent phase-resolved ground truth. The demonstrated applicability and reported performance are limited to 2205 duplex stainless steel aged at 850 °C and imaged at 5000× using the acquisition protocol described here. Transfer to other materials or imaging domains requires separate validation and may require image recalibration, model fine-tuning, transfer learning, or complete retraining.

• The workflow provides a reproducible route from raw SEM/BSE images to binary masks and particle-level descriptors.
• Image-level splitting and fixed inference settings reduce data leakage and post-hoc condition tuning.
• The method preserves compatibility with conventional area fraction, particle count, particle size, and morphology measurements

## Graphical abstract

**Figure:**
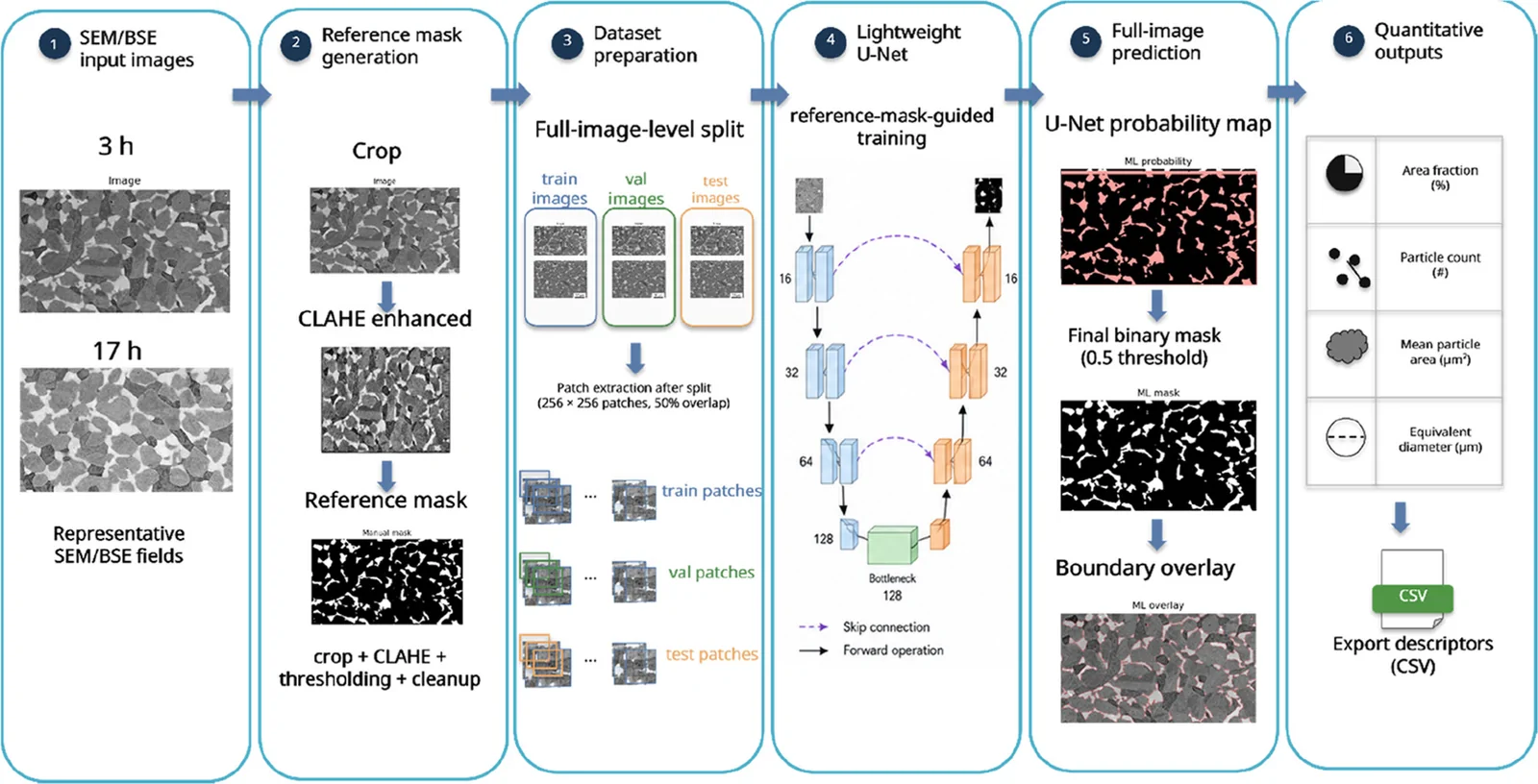

## Specifications table

**Table:** 

**Subject area**	Materials Science
**More specific subject area**	SEM/BSE microstructure image analysis; supervised segmentation; stainless steel; deleterious particle quantification; thermally aged 2205 duplex stainless steel
**Name of your method**	Reference-mask-guided U-Net segmentation and connected-component quantification
Name and reference of original method	The method applies a compact U-Net configuration to grayscale SEM/BSE bright-particle segmentation and integrates it with a previously developed Python-based image-analysis workflow based on contrast enhancement, threshold-derived reference-mask generation, and connected-component quantification. The methodological contribution lies in the reproducible integration of these stages rather than in the development of a new segmentation architecture. The methodological contribution lies in the reproducible integration of these stages rather than in the development of a new segmentation architecture. Key methodological references are: Ronneberger, O., Fischer, P., & Brox, T. (2015). U-Net: Convolutional Networks for Biomedical Image Segmentation. arXiv:1505.04597. Ibrahim, I. M., Will, G., Wang, X., Clegg, R., & Bell, S. (2025). Development of a novel deleterious phases analysis and detection method for stainless steel. Materials Characterization, 228, 115363. https://doi.org/10.1016/j.matchar.2025.115363. van der Walt, S., Schönberger, J. L., Nunez-Iglesias, J., Boulogne, F., Warner, J. D., Yager, N., Gouillart, E., Yu, T., & the scikit-image contributors. (2014). scikit-image: image processing in Python. PeerJ, 2, e453. https://doi.org/10.7717/peerj.453.
Resource availability	Code and example data for a minimal reproducible demonstration of the workflow are available at: [https://github.com/IbrahimMohamed94/sem-bse-bright-particle-unet-workflow]. The repository includes representative SEM/BSE images from two ageing conditions, processing scripts, a batch configuration file, software requirements, and a README file describing the workflow. The example dataset is provided to demonstrate the processing structure, reference-mask generation, patch extraction, model-training workflow, prediction workflow, and descriptor-output organisation. It is not intended to reproduce the complete training dataset or the full validation results reported in the companion research article. The complete raw-image dataset and full trained model outputs may be made available from the corresponding author upon reasonable request, subject to data-sharing permissions.

## Background

Backscattered electron scanning electron microscopy is commonly used to examine thermally aged stainless steels because compositional contrast can make secondary particles enriched in heavier alloying elements appear brighter than the surrounding matrix. In duplex stainless steels, these bright regions can provide useful image-based indicators of deleterious Cr- and Mo-rich precipitate evolution, particularly when imaging conditions are kept consistent across a controlled ageing sequence. Once segmented, these regions can be quantified using descriptors such as area fraction, particle count, particle density, size, morphology, and spatial distribution. For the same 2205 duplex stainless steel ageing series used to demonstrate the present workflow, the relationship between microstructural evolution and mechanical degradation was investigated separately using small punch testing, revealing progressive losses in load response, ductility and absorbed energy and increased embrittlement risk [1,2].

A practical challenge is that bright-particle segmentation is rarely constant across all ageing conditions. During thermal exposure, the target regions may evolve from sparse early-stage particles to fragmented intermediate features and eventually to coarser or more connected networks. These changes affect local contrast, boundary definition, particle connectivity, and the performance of conventional threshold-based segmentation. Although manual or condition-specific thresholding can produce useful reference masks, repeating this process across large image datasets is time-consuming and may introduce operator-dependent variation.

The method described here was developed to make bright-particle quantification more reproducible and scalable while retaining the interpretability of conventional image analysis [3]. The workflow first generates reference masks from a defined preprocessing and labeling procedure, then trains a lightweight U-Net model to reproduce that specific segmentation target from grayscale SEM/BSE image patches [4]. The model is therefore evaluated for consistency with the supplied labelling protocol rather than for independent determination of phase-resolved microstructural ground truth. The trained model is used as a segmentation engine, while the final microstructural measurements are extracted using transparent connected-component analysis. This means that the method does not treat the neural network as an independent phase-identification tool. Instead, it uses supervised learning to reproduce an image-based segmentation target defined by the researcher. The distinction matters for materials characterisation, bright SEM/BSE regions can indicate Cr/Mo-rich particles, but assigning every segmented object to a crystallographic phase requires complementary chemical or crystallographic evidence such as EDS, EBSD, or XRD [5,6]. The workflow is therefore best described as reference-mask-guided bright-region segmentation rather than direct sigma-phase mapping.

Recent materials-micrograph segmentation studies have explored more complex alternatives to the original U-Net. These include attention-guided and dilated U-Net ensembles, recurrent residual U-Net variants incorporating squeeze-and-excitation and feature-pyramid processing, DeepLabV3+ models using pretrained feature extractors, and microscopy-specific pretrained encoders [[7], [8], [9], [10]]. Such approaches may provide stronger multiscale feature extraction, attention-based feature selection, or transfer-learning capability. The present method addresses a narrower engineering objective: providing a compact and reproducible route from SEM/BSE images to reference-guided segmentation masks and conventional quantitative descriptors. It does not propose a new segmentation architecture or claim state-of-the-art performance relative to these more complex alternatives.

This article provides the practical steps required to reproduce the workflow, including image preparation, reference-mask generation, dataset splitting, patch extraction, U-Net training, inference, mask reconstruction, connected-component quantification, and quality-control outputs. The workflow has been demonstrated using 2205 duplex stainless steel aged at 850 °C and imaged at 5000× under one consistent preparation and acquisition protocol. Its procedural structure may be adapted to other SEM/BSE datasets where a suitable image-based target can be defined. However, neither the trained model nor its reported performance should be assumed to transfer directly to another alloy, ageing temperature, magnification, microscope, or acquisition setting without separate evaluation.

### Method details

1.Overview of the workflow.

The method consists of a reproducible image-analysis and supervised-segmentation workflow for quantifying bright particle regions in SEM/BSE micrographs. The workflow starts with raw SEM/BSE images and ends with binary predicted masks, visual quality-control overlays, and quantitative particle descriptors exported as CSV files. The method was designed to preserve the interpretability of conventional image analysis while improving the scalability of mask generation across ageing conditions where particle contrast, size, morphology, and connectivity may change.

The full workflow contains the following stages:

1. Acquisition of SEM/BSE micrographs under consistent imaging conditions.
2. Image cropping to remove the SEM footer, scale bar, and non-microstructural regions.
3. Grayscale preprocessing using contrast-limited adaptive histogram equalisation.
4. Generation of reference bright-particle masks using condition-aware thresholding and morphological cleanup.
5. Full image level splitting into training, validation, and unseen test sets.
6. Extraction of overlapping image and mask patches.
7. Training of a lightweight U-Net model to reproduce the reference masks.
8. Reconstruction of full-image predicted masks during inference.
9. Connected-component labelling and extraction of particle descriptors.
10. Generation of quality-control outputs, including probability maps, binary masks, and boundary overlays.

The benefit of the workflow arises from the controlled connection between its stages rather than from any single processing operation. Cropping removes scale bars, microscope footers, and labels that could otherwise be interpreted as bright microstructural features. CLAHE then improves local contrast while limiting excessive amplification of image noise, supporting more consistent identification of weak and spatially variable bright regions. The reference-mask procedure converts this interpretation into a defined and traceable labelling target using recorded intensity thresholds, physical area filters, and quality-control overlays.

The lightweight U-Net is consequently trained to reproduce this defined segmentation target across the ageing conditions represented in the training dataset. Once trained, the model applies one fixed inference threshold and consistent post-processing settings to all new images within the investigated domain, avoiding repeated selection of condition-specific intensity thresholds during routine analysis. Finally, connected-component labelling converts each predicted binary mask into conventional measurements, including bright-particle area fraction, particle count, particle density, size, and morphology. Because these measurements remain linked to explicit segmented regions, they can be checked directly using probability maps and boundary overlays rather than being produced as vague end-to-end property predictions.

Reproducibility is further supported by full-image data splitting before patch extraction, saved split files, recorded scale calibration, fixed preprocessing and inference settings, documented model configurations, and standardised numerical and visual outputs. On the held-out test micrographs, the workflow achieved a Dice coefficient of 0.917, an IoU of 0.854, and an area-fraction MAE of 0.547 percentage points relative to the threshold-derived reference masks. These values demonstrate consistent reproduction of the defined labelling procedure within the investigated material and imaging domain; they should not be interpreted as independent validation of crystallographic phase boundaries.

A key requirement is that data splitting must be performed at the full-image level before patch extraction. Patches extracted from the same SEM/BSE image are highly correlated. Therefore, allowing patches from one image to appear in both training and testing would lead to data leakage and overestimate model performance.

2.Input data requirements.

The method requires SEM/BSE micrographs in which the target features appear as bright regions relative to the surrounding matrix. The images should preferably be collected using consistent microscope settings, including detector mode, accelerating voltage, working distance, magnification, image resolution, and polishing condition. Consistency is important because the trained model learns the relationship between grayscale contrast, local morphology, and the reference bright-particle labels. Recommended input files include raw or exported SEM/BSE images, a metadata table listing sample condition and image ID, a scale calibration value in um/pixel, and a condition label for each image.

For each image, the following information should be recorded:

• sample or ageing condition;
• image identifier;
• pixel size or scale calibration in µm/pixel;
• magnification;
• microscope and detector mode;
• cropping rule used to remove non-microstructural regions;
• any exclusion notes, such as poor focus, charging, contamination, or missing fields of view.

The model described in this article was developed using SEM/BSE micrographs of 2205 duplex stainless steel aged at 850 °C for 6 min, 30 min, 1 h, 3 h, and 17 h. Images were acquired at 5000× using a Phenom XL2 microscope operated in BSE mode at 20 kV and a working distance of approximately 7 mm. Specimen preparation and acquisition settings were kept consistent throughout the dataset. The held-out test set therefore assesses performance on previously unseen micrographs within this material and imaging domain; it does not constitute external validation across different materials, microscopes, or acquisition protocols. These inputs serve different functions within the workflow. Consistent image format and acquisition settings reduce non-microstructural variation in intensity, contrast, and spatial resolution, thereby reducing the risk that the U-Net learns differences associated with the microscope or image-export procedure rather than the intended bright-particle contrast and morphology. Image identifiers and metadata maintain traceability between each micrograph, its reference mask, sample condition, and assigned data split, while also allowing images affected by charging, poor focus, contamination, or changed acquisition settings to be identified. Scale calibration converts the physical object-removal and hole-filling criteria of 0.10 µm² and 0.02 µm², respectively, into the appropriate pixel areas and is required to report particle area, equivalent diameter, and particle density in physical units. Incorrect or inconsistent calibration would therefore affect both reference-mask generation and the resulting quantitative descriptors. Condition labels allow dataset composition and segmentation performance to be examined across the different ageing morphologies; their use during data splitting is described in Section 8.The image target should be defined before training. It should not be changed after training begins, because the model will reproduce the labelling rule it is given. In this method, the target is the bright SEM/BSE particle region. These regions are treated as image-based indicators of compositionally distinct deleterious particles. The procedure for generating reference masks from these regions is detailed in Section 6.

3 Suggested folder structure.

A clear folder structure improves reproducibility. The recommended project layout is shown below: project_root/. data/.

raw_images/.

reference_masks/.

splits/.

patches/.

train/.

val/.

test/.

models/.

outputs/.

predictions/.

overlays/.

metrics/.

descriptors/.

scripts/.

README.md.

The names of image files and masks should match exactly except for a suffix, for example image_001.png and image_001_mask.png. This reduces the risk of pairing the wrong image and mask during training.

4 Image cropping.

Each SEM/BSE micrograph should first be cropped to remove the microscope footer, scale bar, labels, and any non-microstructural regions. These regions should not be included in the model input or the reference mask because they may introduce artificial bright features that are unrelated to the material microstructure. Bright footer text, labels, and scale-bar pixels can exceed the intensity threshold used to generate the reference masks and may therefore be incorrectly labelled as bright-particle regions. Because these features often occur at similar positions across images acquired using the same export format, a supervised model may learn their repeated shapes and locations instead of relying only on microstructural contrast and particle morphology. If retained during connected-component analysis, they would also increase the segmented area and could distort particle count, particle size, morphology, and density measurements. Cropping must therefore be completed before reference-mask generation and patch extraction, with the identical crop applied to each image and its corresponding mask to preserve pixel-level alignment. Removing the displayed scale bar does not remove the spatial calibration because the numerical pixel-to-micrometre value is recorded separately in the image metadata and used during quantitative analysis.

In the present workflow, a fixed bottom crop of 75 pixels was applied to each SEM/BSE image to remove the footer region. The same crop was applied to the grayscale image and the corresponding reference mask. If images are acquired using a different microscope, export format, or magnification, the crop value should be adjusted after visual inspection and recorded in the metadata file.

The pixel-to-micrometre calibration should be recorded before quantitative descriptor extraction. All physical area-based filters and descriptors depend on this calibration. The calibration value should be used consistently when converting pixel area into µm² and when calculating descriptors such as particle area, equivalent diameter, and particle density.

Before proceeding, the following checks should be performed (quality control check):

• The cropped image contains only the microstructure.
• The same crop has been applied to the image and mask.
• The scale calibration is correct for the cropped image.
• No scale bar, text, or footer artefact remains in the analysed region.

5 Contrast enhancement.

The cropped SEM/BSE image is converted to grayscale if it is not already stored as a single-channel image. Contrast-limited adaptive histogram equalisation is then applied to improve local contrast between bright particle regions and the surrounding matrix [11]. This step is useful when the target particles are visible but the local background intensity varies across the image.

Suggested starting settings are a CLAHE clip limit of 2.0 and a tile grid size of 8 × 8. These values are not universal and should be checked visually using representative images from each ageing condition.

These values were selected to enhance local bright-particle contrast without excessively amplifying noise. The same CLAHE settings should be used consistently for all images in the dataset unless a separate optimisation step is reported. If the settings are changed for another dataset, the new values should be recorded in the method metadata and applied consistently to both reference-mask generation and model input preparation. The preprocessed image should be inspected visually before thresholding. Over-enhancement can create artificial bright spots, while insufficient enhancement can cause weak particle boundaries to be missed.

6 Reference bright-particle mask generation.

Reference masks are generated from the cropped and contrast-enhanced SEM/BSE images. These masks define the segmentation target that the U-Net will learn. Therefore, the reference-mask workflow must be fixed before training and applied consistently within each condition. Each operation controls a different potential source of inconsistency in the training labels. Cropping excludes non-microstructural bright regions, while CLAHE improves local particle-to-matrix contrast using fixed enhancement settings. The predetermined intensity threshold then converts this enhanced contrast into an explicit foreground-selection rule. Subsequent removal of isolated noise and filling of small internal holes improve the continuity of the selected regions before the final mask is encoded as background = 0 and bright particle = 1 for supervised training. Standardisation in this context refers to applying the same documented processing sequence and predetermined parameters within each condition, rather than imposing one universal intensity threshold on micrographs with different contrast characteristics. This approach avoids image-by-image post-hoc adjustment, reduces operator-dependent label variation, and provides the U-Net with a more consistent segmentation target. It improves agreement with the defined reference-mask workflow but does not independently establish crystallographic phase identity.

The reference-mask generation procedure consists of the following steps:

1. crop the SEM/BSE image to remove non-microstructural regions;
2. apply CLAHE to the cropped grayscale image;
3. segment bright regions using an intensity-greater-than threshold;
4. remove small objects below the selected minimum physical area to reduce isolated noise;
5. fill small holes within segmented bright regions to avoid any artificial voids inside segmented bright regions;
6. save the final binary mask using the same image identifier as the original micrograph.

Because bright-region contrast and morphology may change with ageing condition, condition-specific intensity thresholds can be used when justified by visual inspection and threshold-sensitivity testing. In the present dataset, Table 1 shows the thresholds which were used.

**Table 1:** Thresholds used for the present dataset.

**Ageing condition**	**Intensity threshold rule**
6 min	intensity > 235
30 min	intensity > 225
1 h	intensity > 190
3 h	intensity > 180
17 h	intensity > 190

After thresholding, objects smaller than 0.10 µm² were removed to reduce isolated noise. Small holes below 0.02 µm² were filled to avoid artificial voids inside segmented bright regions. These size filters were applied in physical units using the image scale calibration.

Expressing these criteria in physical units ensures that their effective size does not change solely because of image resolution or pixel dimensions. For a pixel size of (s) µm/pixel, the corresponding pixel-area criterion is calculated from (A/s^2^), where (A) is the specified physical area. The 0.10 µm² minimum-object criterion removes isolated high-intensity specks and very small connected regions that are more likely to represent image noise than reliably measurable particle features. Without this filter, these regions could disproportionately increase particle count and density and distort the lower end of the particle-size distribution. This value also defines an operational lower measurement cutoff: regions smaller than 0.10 µm² are excluded and the results should not be interpreted as quantifying particles below this area. The 0.02 µm² hole-filling criterion removes only small enclosed background gaps within segmented bright regions, thereby limiting artificial fragmentation and underestimation of particle area. Applying both criteria consistently produces cleaner and more topologically stable reference masks for supervised training. These values were fixed for the present dataset and should be re-evaluated and reported when the method is applied at a different image scale or resolution.

The final masks should be interpreted as reference bright-particle masks, not as independent crystallographic phase maps. The labels represent bright SEM/BSE regions selected by a reproducible image-analysis rule. Any phase-level interpretation, such as assigning the regions to sigma or chi phase, should be supported by complementary evidence such as EDS, EBSD, XRD, or other correlative characterisation. Because the reference masks are derived from intensity thresholding, their boundaries may remain uncertain where BSE contrast is weak, where adjacent bright regions are connected, or where polishing and imaging artefacts alter the local grayscale intensity. A supervised model trained using these masks cannot be assumed to correct systematic uncertainty already present in the labels. A local difference between a prediction and its reference mask may therefore arise from model error, uncertainty in the reference boundary, or a combination of both.

7 Reference-mask quality control.

Each reference mask should be checked using a boundary overlay on the original SEM/BSE image. This step is important because errors in the reference masks will be learned by the U-Net.

For each image, save at least the following quality-control files:

• cropped grayscale image.
• CLAHE-enhanced image.
• binary reference mask.
• reference-mask boundary overlay.

The overlay should be inspected to confirm that the mask captures the intended bright-particle regions without including polishing scratches, contamination, scale-bar remnants, footer text, or background contrast artefacts.

Images should be excluded or flagged if they show:

• Poor focus.
• Charging artefacts.
• Contamination.
• Severe brightness/contrast inconsistency.
• Incomplete fields of view.
• Footer or scale-bar remnants after cropping.
• Missing or incorrect scale calibration.

This quality-control step is essential because the model can only be as reliable as the reference masks used for training.

8 Data splitting.

The dataset should be split at the full-image level before patch extraction. This is a critical step because neighbouring patches extracted from the same SEM/BSE image are strongly correlated. If patches from one image are allowed to appear in both the training and test sets, the reported model performance may be artificially high due to data leakage. To maintain representation across the ageing sequence, the present dataset was divided within each ageing condition before the condition-specific subsets were combined. Consequently, the training, validation, and test sets each contained micrographs from all five ageing conditions, while every extracted patch retained the split assigned to its parent micrograph. The condition labels were used only for dataset organisation, split verification, and condition-level evaluation; they were not supplied to the U-Net as predictive inputs.

Recommended split files are train_images.csv, val_images.csv, and test_images.csv. Each file should include image ID, condition, raw image path, reference mask path, pixel size, and notes or exclusion flags.

The unseen test set should remain untouched during model development. It should not be used for threshold selection, model selection, probability-threshold tuning, post-processing decisions, or visual correction of the workflow.

The training, validation, and test subsets serve different purposes. The training data are used to update the model weights, whereas the validation data provide independent feedback during model development without contributing to gradient-based weight updates. Validation performance can therefore be used to select the best checkpoint and, where applicable, to compare hyperparameters, probability thresholds, or post-processing settings. However, because these development decisions are progressively informed by the validation results, validation performance should not be treated as the final estimate of generalisation. The test set must remain outside this decision process and should be evaluated only after the architecture, model weights, probability threshold, and post-processing rules have been fixed. Otherwise, information from the test images indirectly influences the workflow, producing data leakage and an optimistically biased estimate of performance. In the present workflow, the checkpoint with the highest validation Dice coefficient was selected, while the probability threshold of 0.5 and the absence of morphological closing were fixed without reference to the unseen test results.

9.Patch extraction.

Full SEM/BSE images are divided into smaller patches for U-Net training. A patch size of 256×256 pixels is suitable for the lightweight U-Net described here. A stride of 128 pixels provides 50% overlap, which increases the number of training samples and reduces edge artefacts.

Recommended settings: patch size 256×256 pixels; stride 128 pixels; image patches as grayscale arrays normalised to a consistent intensity range; mask patches as binary arrays with background = 0 and bright particle = 1.

The extracted patches must remain in the same dataset split as their parent image. For example, patches derived from a training image should only be saved in the training patch folder, and patches derived from an unseen test image should only be saved in the test patch folder.

A suitable naming convention is:

DSS_ 1h_img_001_x0000_y0000.png.

DSS_ 1h_img_001_x0128_y0000.png.

DSS_ 1h_img_001_x0256_y0000.png.

For the corresponding masks:

DSS_ 1h_img_001_x0000_y0000_mask.png.

DSS_ 1h_img_001_x0128_y0000_mask.png.

DSS_ 1h_img_001_x0256_y0000_mask.png.

This naming convention preserves the parent image identifier and patch coordinates, allowing each patch to be traced back to its original SEM/BSE micrograph.

Patches with no bright pixels may be retained because they teach the model background features. However, if the dataset is highly imbalanced, the number of empty patches can be controlled so training is not dominated by background-only examples.

10 U-Net architecture.

A lightweight U-Net was used for binary segmentation of bright-particle regions from grayscale SEM/BSE image patches. The model input was a single-channel 256 × 256 pixel image patch, and the output was a single-channel probability map of the same spatial size. Each output pixel represented the predicted probability of belonging to the bright-particle class.

The network followed an encoder-decoder structure with skip connections. The encoder extracted local and contextual image features, while the decoder reconstructed the segmentation map at the original patch resolution. Skip connections were used to transfer spatial detail and features from the encoder to the corresponding decoder stage, which is important for recovering fine bright-particle boundaries.

The architecture used the following channel progression: input channels = 1. encoder channels = 16, 32, 64. bottleneck channels = 128. decoder channels = 64, 32, 16. output channels = 1.

Recommended architecture (used): input 1 × 256×256 grayscale patch; encoder channels 16, 32, and 64; bottleneck channels 128; decoder channels 64, 32, and 16; convolution block with two 3 × 3 convolutions, each followed by batch normalisation and ReLU activation; 2 × 2 max-pooling for downsampling; transposed convolution for upsampling; skip connections between matching encoder and decoder levels; final 1 × 1 convolution to one output channel; sigmoid activation during inference.

Each encoder block consisted of two 3 × 3 convolution layers, with each convolution followed by batch normalisation and ReLU activation. Downsampling was performed using 2 × 2 max pooling. The decoder used transposed convolutions for upsampling, followed by concatenation with the corresponding encoder feature map. A final 1 × 1 convolution was used to generate the single-channel segmentation output.

A lightweight architecture was selected because SEM/BSE datasets are often much smaller than natural-image datasets. The reduced channel depth helped limit overfitting while still providing enough capacity to learn bright-particle contrast, boundary morphology, and connected network features.

The lightweight U-Net contained 483,153 trainable parameters, and the saved model checkpoint occupied 5.62 MB. In the CPU-only implementation used for the companion evaluation, a complete 40-epoch training run required approximately 4.52 h. End-to-end processing, including image loading, patch extraction, prediction, full-image reconstruction, post-processing, connected-component quantification, and output saving, required an average of 3.476 s per full micrograph. These values are specific to the hardware and software environment used in this study, but they show that the selected architecture can be trained and deployed without specialised GPU hardware.

To provide a controlled internal comparison, the lightweight U-Net was evaluated against a supervised pixel-level Random Forest and a validation-tuned global threshold. The Random Forest was trained using the same full-image training set and threshold-derived reference masks as the U-Net, while its probability threshold was selected using only the validation set. All three approaches were evaluated on the same held-out test micrographs using consistent post-processing and quantitative metrics. The lightweight U-Net achieved a Dice coefficient of 0.917 and an area-fraction MAE of 0.547 percentage points. The supervised Random Forest achieved a Dice coefficient of 0.797 and an area-fraction MAE of 1.034 percentage points, while the validation-tuned global threshold achieved a Dice coefficient of 0.707 and an area-fraction MAE of 2.876 percentage points. These comparisons support the lightweight U-Net as an effective engineering choice relative to the simpler supervised and intensity-based baselines examined under controlled conditions. They do not establish superiority over contemporary deep-learning segmentation architectures.

Attention-guided U-Nets, nested U-Net variants, DeepLabV3+, and transformer-based segmentation models remain reasonable candidates for future controlled benchmarking. They were not trained in the present Methods study because the objective was to document and validate the complete reference-mask-guided workflow rather than optimise the segmentation architecture. A fair comparison would require all candidate models to be trained using the same full-image splits, reference masks, validation-based model-selection procedure, inference threshold, post-processing rules, and repeated random seeds. Published performance values obtained using different materials, imaging modalities, targets, labels, and data partitions cannot provide such a controlled comparison. The selected lightweight U-Net should therefore be interpreted as an adequate and computationally practical configuration for the demonstrated workflow, not as an optimal or universally superior segmentation architecture.

11 Model training.

The U-Net model was trained to reproduce the reference bright-particle masks generated from the image-analysis workflow. The model was implemented using PyTorch [12]. The task was treated as binary semantic segmentation, where background pixels were labelled as 0 and bright-particle pixels were labelled as 1. The model was trained using a combined binary cross-entropy and Dice loss. Binary cross-entropy supports pixel-wise classification, while Dice loss improves overlap between the predicted and reference masks. This combination is useful for bright-particle segmentation because the bright-particle class may occupy a smaller area than the background, especially in early ageing conditions.

Recommended training settings: optimiser AdamW; learning rate 0.001; batch size 4; 40 epochs; loss function binary cross-entropy plus Dice loss; model selection based on best validation Dice coefficient.

During training, the validation set was used only for monitoring model performance and selecting the best model weights. The unseen test set was not used during training, hyperparameter selection, probability-threshold selection, or post-processing decisions.

Training outputs should include training loss per epoch, validation loss per epoch, training Dice coefficient per epoch, validation Dice coefficient per epoch, saved best model weights, and final training configuration file.

The training configuration file should include the patch size, stride, input normalisation method, loss function, optimiser, learning rate, batch size, number of epochs, random seed, and paths to the split files. Saving this information is important because model weights alone are not sufficient to reproduce the workflow.

12 Inference and mask reconstruction.

During inference, the trained U-Net produces a probability map for each image patch. The raw model output is converted into probabilities using a sigmoid function. A fixed probability threshold is then applied to convert the probability map into a binary mask. In this workflow a probability threshold of 0.5 is applied to generate the binary predicted mask.

The same probability threshold should be used for all ageing conditions unless a separate calibration experiment is explicitly reported. Avoid condition-specific post-hoc thresholding because it reduces the practical value of the supervised model.

For full-image prediction, patches are predicted individually and then reconstructed into the original image coordinate system. When overlapping patches are used, the predicted probabilities in overlapping regions should be averaged before applying the final binary threshold. Averaging overlapping probabilities helps reduce patch-edge artefacts.

Recommended outputs include the predicted probability map, binary predicted mask, boundary overlay on the original SEM/BSE image, image-level metrics CSV file, and particle-level descriptor CSV file.

No additional morphological closing was applied to the predicted masks in the core workflow. This was done to avoid artificially merging adjacent bright regions, which can affect object-level descriptors such as particle count and mean particle area.

If post-processing is added for another dataset, it should be reported explicitly. The same post-processing rule should be applied consistently to all images, and its effect on both area fraction and object-level descriptors should be checked.

13 Connected-component quantification.

After the full-image binary mask is generated, connected-component labelling is applied to identify individual bright-particle regions using standard Python image-analysis operations [13]. Each connected bright region is treated as one object. The same connected-component analysis should be applied to both reference masks and U-Net-predicted masks so that descriptor values are directly comparable.

The binary mask is first converted into labelled regions. Background pixels are assigned a value of **0**, while each connected bright region is assigned a unique object label. Particle descriptors are then extracted from each labelled region.

Image-level descriptors include bright-particle area fraction, total bright area, particle count, particle density, mean particle area, median particle area, and mean equivalent diameter.

The following image-level descriptors can be exported for each SEM/BSE micrograph:

• bright-particle area fraction (%).
• total bright-particle area (µm²).
• particle count.
• particle density (particles/mm² or particles/µm²).
• mean particle area (µm²).
• median particle area (µm²).
• mean equivalent diameter (µm).
• median equivalent diameter (µm).

Particle-level descriptors include area, centroid, perimeter, equivalent diameter, major axis length, minor axis length, aspect ratio, circularity, solidity, eccentricity, orientation, and mean grayscale intensity if required.

The following particle-level descriptors can be exported for each labelled bright region:

• particle ID.
• image ID.
• ageing condition.
• area (µm²).
• centroid x position.
• centroid y position.
• perimeter.
• equivalent diameter.
• major axis length.
• minor axis length.
• aspect ratio.
• circularity.
• solidity.
• eccentricity.
• orientation.
• mean grayscale intensity.

Area-based descriptors should be converted from pixels to physical units using the recorded pixel-size calibration. For example, particle area can be calculated as: particle area (µm²) = particle area (pixels) × (pixel size in µm/pixel)²

Equivalent diameter can then be calculated from the particle area assuming a circle of equal area: equivalent diameter = 2 × √(particle area / π).

Circularity can be calculated as: circularity = 4π × area / perimeter²

A circularity value closer to 1 indicates a more circular object, while lower values indicate elongated, irregular, or connected features.

Bright-particle area fraction is usually the most robust descriptor because it depends mainly on the total segmented bright area. In contrast, object-level descriptors such as particle count, mean particle size, and particle density are more sensitive to local splitting and merging of connected regions.

For example, a thin bridge between two adjacent bright particles can convert two labelled objects into one object without strongly changing the total area fraction. Similarly, a small missed boundary segment can change the particle count while producing only a small area-fraction difference. For this reason, object-level descriptors should be interpreted together with boundary overlays and condition-level trends rather than as isolated values.

14 Output files.

The workflow should generate both visual and numerical outputs. The recommended numerical outputs are:

• image_level_descriptors.csv.
• particle_level_descriptors.csv.
• model_evaluation_metrics.csv.
• training_history.csv.

The image_level_descriptors.csv file should contain one row per SEM/BSE image. Suggested columns are:

• image_id.
• condition.
• split.
• area_fraction_percent.
• total_bright_area_um2.
• particle_count.
• particle_density.
• mean_particle_area_um2.
• median_particle_area_um2.
• mean_equivalent_diameter_um.
• median_equivalent_diameter_um.
• notes.

The particle_level_descriptors.csv file should contain one row per labelled bright-particle object. Suggested columns are:

• image_id.
• condition.
• particle_id.
• area_um2.
• centroid_x_px.
• centroid_y_px.
• centroid_x_um.
• centroid_y_um.
• perimeter_um.
• equivalent_diameter_um.
• major_axis_length_um.
• minor_axis_length_um.
• aspect_ratio.
• circularity.
• solidity.
• eccentricity.
• orientation.
• mean_intensity.

The visual outputs should include:

• cropped SEM/BSE image.
• CLAHE-enhanced image.
• reference mask.
• predicted probability map.
• predicted binary mask.
• reference boundary overlay.
• predicted boundary overlay.
• side-by-side comparison panel.

These outputs are useful for checking whether the model is segmenting the intended bright-particle regions and whether the descriptor values are physically reasonable.

15 Quality control outputs.

Quality control is essential for reproducibility. For each image, the workflow should save an overlay showing predicted mask boundaries on the original SEM/BSE micrograph. This allows the user to identify false positives, missed thin features, excessive boundary thickening, or object merging.

Recommended checks include:

1. confirm that area fraction values are within a physically reasonable range.
2. check images with unusually high or low particle counts.
3. inspect overlays for images with high particle-density values.
4. verify that large connected networks are not incorrectly interpreted as many small particles.
5. confirm that scale calibration was applied consistently.
6. compare reference and predicted descriptors for validation and test images.

Images with poor focus, charging artefacts, contamination, inconsistent contrast, or incorrect cropping should be flagged and excluded or analysed separately. Any excluded or flagged image should be recorded in the metadata file with a short explanation. This improves reproducibility and makes it clear whether exclusion was based on image quality, missing data, or segmentation failure.

## Method validation

Model evaluation should be performed using complete micrographs that were not used during training, validation-based model selection, probability-threshold selection, or post-processing decisions. In the present application, the U-Net predictions were compared with threshold-derived reference masks on a held-out test set. Accordingly, the reported Dice, intersection over union, precision, and recall values are measures of agreement with the supplied reference masks rather than estimates of agreement with independent phase-resolved ground truth. The complete model-development, comparative-segmentation, and condition-level results are reported in the companion research article [1].

Evaluation was performed at two levels. Pixel-level agreement was used to determine how closely the predicted binary masks reproduced the spatial regions defined by the reference-mask procedure. Descriptor-level agreement was then used to determine whether the predicted and reference masks produced similar measurements after the same connected-component analysis was applied.

Representative EDS point measurements supported the interpretation of selected bright BSE features as Cr/Mo-rich regions. However, these measurements were not spatially registered phase maps and were not used as pixel-level labels. Spatially matched EDS or EBSD maps and independently prepared expert masks were not available for the present evaluation. The EDS evidence therefore provides metallurgical context for the selected image target but does not constitute independent validation of individual segmentation boundaries.

The recommended pixel-level validation metrics are:

• Dice coefficient.
• intersection over union.
• precision.
• recall.

The recommended descriptor-level validation metrics are:

• bright-particle area-fraction error.
• area-fraction bias.
• particle-count error.
• mean particle-area error.

**Fig. 1:**
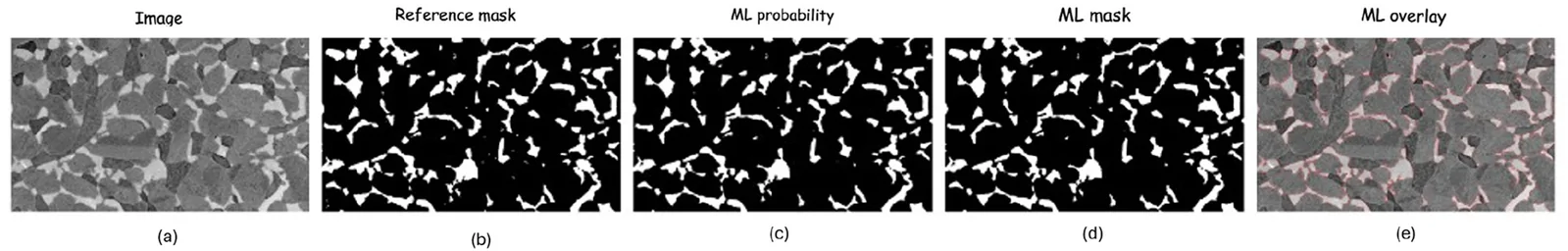
Example validation output from an unseen SEM/BSE test image of 2205 duplex stainless steel aged at 850 °C for 3 h. (a) Cropped SEM/BSE micrograph, (b) reference bright-particle mask generated using the image-analysis workflow, (c) U-Net probability map, (d) final binary U-Net mask after fixed probability thresholding, and (e) predicted bright-particle boundary overlay on the original SEM/BSE image.

Fig. 1 shows an example validation output from an unseen test image. The figure illustrates the full output sequence produced by the workflow: the cropped SEM/BSE image, the reference bright-particle mask, the U-Net probability map, the final binary U-Net mask, and the predicted boundary overlay. The overlay is used as a visual quality-control step to confirm that the model segments the intended bright-particle regions rather than polishing artefacts, matrix contrast, or non-microstructural features.

The Dice coefficient and intersection over union measure spatial overlap between the reference and predicted masks. Precision indicates the proportion of predicted bright-particle pixels that were also labelled as bright particles in the reference mask. Recall indicates the proportion of reference bright-particle pixels recovered by the model.

To complement the quantitative agreement metrics, a qualitative audit was conducted using one prespecified held-out micrograph from each of the five ageing conditions. The same image index was used across the conditions so that the examples were not selected according to their apparent prediction quality. The original BSE image, threshold-derived reference mask, predicted probability map, final binary mask, and boundary overlay were examined together. Most disagreements were localised around very small or weak bright features, thin fragmented regions, and the boundaries of connected bright-particle networks. This audit was used to identify characteristic disagreement modes and was not used to modify the reference masks, adjust the model, or select inference settings. It also cannot determine whether an individual disagreement represents model error or uncertainty in the threshold-derived reference boundary.

The overall validation results are summarised in Table 2 Pixel-level metrics were used to assess mask agreement, while descriptor-level metrics were used to assess whether the predicted masks produced comparable quantitative microstructural measurements. The main descriptor of interest was bright-particle area fraction, because it depends primarily on the total segmented bright area and is less sensitive to local object splitting or merging than particle count.

**Table 2:** Agreement between U-Net predictions and threshold-derived reference masks on held-out test micrographs.

**Validation output**	**Metric**	**Value**	**Interpretation**
Pixel-level mask agreement	Dice coefficient	0.917	Measures spatial overlap between predicted and threshold-derived reference masks
Pixel-level mask agreement	Intersection over union	0.854	Measures the shared predicted and reference area relative to their combined area
Descriptor-level agreement	Area-fraction MAE	0.547 pp	Mean absolute difference from the area fraction assigned by the reference-mask procedure
Descriptor-level agreement	Area-fraction bias	+0.098 pp	Mean signed difference from the area fraction assigned by the reference-mask procedure
Object-level agreement	Particle-count MAE	26.3 particles	Mean difference in connected component count relative to the reference masks; sensitive to local splitting and merging

To quantify sampling uncertainty in these agreement estimates, a stratified image-level percentile bootstrap was performed using 10,000 repetitions, with each complete test micrograph treated as the independent sampling unit. For the primary model reported in Table 2 the Dice coefficient of 0.917 had a 95% confidence interval of 0.904–0.929, the IoU of 0.854 had an interval of 0.834–0.873, and the area-fraction MAE of 0.547 percentage points had an interval of 0.425–0.678 percentage points. Sensitivity to stochastic model training was assessed separately by repeating the complete training and evaluation procedure using five random seeds while holding the image splits, architecture, training settings, checkpoint-selection procedure, probability threshold, and post-processing rules constant. Across these runs, the mean unseen-test Dice coefficient was 0.919 ± 0.009 and the mean IoU was 0.857 ± 0.014. These analyses indicate that agreement with the threshold-derived reference masks was reasonably stable within the available held-out dataset and was not dependent on a single favourable model initialisation.

On the held-out test micrographs, the U-Net predictions showed close agreement with the threshold-derived reference masks at both the pixel and area-fraction levels. The area-fraction MAE of 0.547 percentage points indicates that the model reproduced the area fractions assigned by the reference-mask procedure with a small mean absolute difference. This value should not be interpreted as an error relative to the true crystallographic phase fraction.

The larger particle-count difference reflects the sensitivity of connected-component measurements to small changes in boundary continuity. Local bridging, fragmentation, or boundary displacement can change the number of labelled objects without producing a comparable change in total segmented area. Area fraction was therefore the most reproducible descriptor relative to the supplied reference masks, whereas particle count and size-related descriptors should be interpreted together with the boundary overlays and condition-level trends.

These results demonstrate that the U-Net consistently reproduced the defined reference-mask procedure within the investigated dataset and imaging conditions. They do not establish phase-specific boundary accuracy. Independent expert annotations or spatially correlated EDS/EBSD labels would be required to determine whether individual predicted boundaries more closely represent the underlying microstructure than the threshold-derived reference boundaries.

In this article, generalisation refers specifically to performance on held-out micrographs from the five ageing durations represented in the dataset. All images were obtained from the same alloy at the same ageing temperature and magnification using a consistent acquisition procedure. The evaluation therefore demonstrates within-domain generalisation across different ageing-stage morphologies, but it does not establish transfer to other alloy grades, ageing temperatures, magnifications, imaging systems, or laboratories.

### Limitations

The proposed workflow is designed for supervised segmentation of bright SEM/BSE particle regions. It should not be interpreted as an independent crystallographic or chemical phase-identification method. The U-Net learns to reproduce the reference masks provided during training; therefore, the segmented regions represent the image-based target defined by the reference-mask generation procedure. Assigning these regions to specific phases, such as sigma or chi phase, requires supporting evidence from complementary characterisation methods such as EDS, EBSD, XRD, or other correlative techniques.

For the present dataset, representative EDS point measurements confirmed Cr/Mo enrichment in selected bright BSE features and supported the metallurgical interpretation of the image target. However, these measurements were not spatially matched phase maps and therefore did not provide independent pixel-level validation of the predicted boundaries. Independently prepared expert masks and spatially correlated EDS or EBSD labels were not available. The reported overlap and descriptor metrics should consequently be interpreted as measures of consistency with the threshold-derived reference-mask procedure.

The trained model and reported performance are specific to the investigated domain: 2205 duplex stainless steel aged at 850 °C, imaged at 5000× using one microscope and a consistent specimen-preparation and acquisition procedure. Changes in alloy composition or ageing temperature may alter the fraction, morphology, connectivity, and BSE contrast of the target features. Changes in magnification affect particle dimensions, boundary detail, and apparent connectivity in pixel space. Differences in detector response, accelerating voltage, working distance, brightness, contrast, image noise, polishing quality, contamination, or charging behaviour may also shift the image-intensity distribution relative to the training data. Any of these changes may reduce segmentation performance.

The quality of the reference masks is another important limitation. If the reference masks contain inconsistent thresholding decisions, polishing artefacts, contamination, or incorrectly labelled matrix contrast, the model may learn these errors. Reference-mask boundary overlays should therefore be inspected before training, and poor-quality images should be excluded or flagged in the metadata.

The workflow is more robust for area-based descriptors than for object-level descriptors. Bright-particle area fraction depends mainly on the total segmented area and is less affected by small local boundary differences. In contrast, particle count, particle density, and size-distribution descriptors are more sensitive to splitting, merging, and boundary-continuity errors during connected-component labelling. These object-level descriptors should be interpreted together with visual overlays and condition-level trends.

Finally, the workflow has been demonstrated using SEM/BSE images from 2205 duplex stainless steel aged at 850 °C and acquired at 5000× under one consistent imaging protocol. The generalisation demonstrated here is therefore limited to held-out micrographs from the five ageing durations included in the present dataset. Application to other alloys, ageing temperatures, imaging magnifications, particle types, etching conditions, microscopes, or acquisition settings may require new reference masks, image-intensity calibration, model fine-tuning, transfer learning, retraining, or additional validation. For broader use, the method should be tested on independently acquired datasets and, where possible, supported by correlative phase-level labels.

## Ethics statements

This work did not involve human subjects, animal experiments, or data collected from social media platforms.

## CRediT author statement

**Ibrahim M. Ibrahim:** Conceptualization, Methodology, Investigation, Visualization, Software, Validation, Formal analysis, Validity tests, Data Curation, Project administration, Writing - Original Draft, Writing & Editing. **Geoffrey Will:** Supervision, Writing - Review & Editing, Resources, Investigation. **Richard Clegg:** Supervision. **Stuart Bell:** Supervision, Writing - Review & Editing, Resources, Investigation.

## Supplementary material and/or additional information [OPTIONAL]

A minimal demonstration package is provided through a GitHub repository: [https://github.com/IbrahimMohamed94/sem-bse-bright-particle-unet-workflow]. The repository includes representative SEM/BSE images from two ageing conditions, processing scripts, a batch configuration file, software requirements, and a README file describing the workflow. The example dataset is provided to demonstrate the processing structure, reference-mask generation, patch extraction, model-training workflow, prediction workflow, and descriptor-output organisation. It is not intended to reproduce the complete training dataset or the full validation results reported in the companion research article.

During method development, several conventional thresholding options were examined, including automatic and percentile-based approaches. These alternatives were useful for checking threshold sensitivity, but they were not adopted as the final scalable workflow because bright-particle contrast and morphology varied between ageing conditions. The final workflow therefore uses reference-mask-guided supervised segmentation followed by connected-component quantification.

## Declaration of competing interest

The authors declare that they have no known competing financial interests or personal relationships that could have appeared to influence the work reported in this paper.

## Data Availability

Code and a reproducible demonstration dataset are openly available at: https://github.com/IbrahimMohamed94/sem-bse-bright-particle-unet-workflow.
